# Application of deep learning in behavior recognition and early warning system for campus safety management

**DOI:** 10.1371/journal.pone.0335040

**Published:** 2025-11-04

**Authors:** Li Liu

**Affiliations:** Nanjing Audit University Jinshen College, Nanjing, Jiangsu, China; University of Sargodha, PAKISTAN

## Abstract

Campus safety is an essential concern as schools, colleges, and universities work to create secure environments for students, staff, and visitors. Many existing security systems are not fully effective at detecting unusual behaviors or sending fast alerts, which can delay responses to potential threats. To improve this, the research introduces DeepCARE(Deep-learning-based Campus Anomaly & Risk Evaluation), a deep learning-based framework designed to enhance behavior recognition and early warning for campus security. DeepCARE combines convolutional neural networks (CNNs) with long short-term memory (LSTM) networks to process real-time video footage and detect abnormal activities such as aggression, unauthorized access, and people staying too long in restricted areas. The system’s main feature is its hybrid model, where CNNs extract key visual features from surveillance footage while LSTM networks analyze these features over time to recognize behavior patterns. DeepCARE also includes an anomaly detection module using autoencoders, which helps improve the system’s accuracy and reduces false alarms. This makes DeepCARE a flexible and scalable solution, suitable not only for educational campuses but also for public spaces, transport hubs, and smart cities. By applying deep learning, DeepCARE supports early risk detection and faster response times, helping security teams create safer spaces. Experimental results show that DeepCARE achieves a behavior recognition accuracy of 94.5%, performs 8% better than traditional methods, and shortens emergency response times by 30%.

## 1. Introduction

Campus safety is a global concern for educational institutions, necessitating effective strategies to safeguard students, staff, and visitors [1]. The complexities of maintaining secure environments demand the ability to detect and respond to diverse security events. Conventional security approaches, often dependent on static surveillance and manual observation, encounter difficulties adapting to the dynamics of campus settings [2].

Intelligent, proactive systems can improve campus security thanks to technology, especially artificial intelligence [3]. Complex pattern recognition and real-time data analysis can improve campus safety management. Frequent security incidents underscore the need for sophisticated security solutions. Current methods have trouble identifying risky behavioral indicators [4]. Human observation can cause inconsistencies and delays, especially on big campuses. Automation and improvement of odd behavior identification are possible using deep learning [5]. This method gives security professionals quick and reliable information for preemptive intervention [6]. Secure campus environments require intelligent technologies that learn and react to changing security threats.

Behavior recognition in video surveillance is possible with deep learning methods like convolutional neural networks (CNNs) [7] and long short-term memory (LSTM) networks [8]. CNNs capture spatial characteristics from visual inputs, while LSTMs evaluate temporal sequences, making them ideal for capturing human behavior’s dynamic nature [9]. These methods allow a comprehensive security system to automatically identify aggressiveness, illicit entrance, and prolonged presence in prohibited areas [10]. Autoencoders and other anomaly detection algorithms improve the system’s ability to detect deviations from predicted behavior patterns, enhancing accuracy.

Deep learning extends beyond behavior detection in campus security. It also involves creating risk-prediction early warning systems [11]. These technologies analyze behavior patterns over time to identify risk indications, allowing security professionals to act [12]. This proactive security technique can improve campus safety and well-being. Developing scalable and adaptive solutions that integrate with security infrastructure is crucial for wider adoption of these technologies.

The motivation arises from the potential of deep learning to enhance security systems. Deep learning models that learn complicated patterns from datasets make behavior recognition possible. GPU-enabled real-time processing allows responsive systems [13]. Combine CNNs and LSTMs to capture dynamic behavior. Autoencoders detect deviations to strengthen systems. Increased surveillance footage and edge device power improve adoption. The smart city movement promotes better security.

Problem Definition: The primary technical challenge is the automated detection of unusual behaviors within real-time video surveillance, providing rapid alerts [14]. Specifically, the challenge involves

1) Reliable Classification of Unusual Behaviors: Normal and abnormal campus activities must be distinguished, including hostility, unauthorized entrance, and lengthy presence. These actions suggest danger, access, or suspicion. These anomalous behaviors must be reliably classified by the system.
2) Optimizing alert accuracy: Security systems should eliminate false positives and negatives to improve efficacy and avoid disturbances. An anomaly detection module uses autoencoders and deep learning to detect deviations from predicted behavior patterns, reduce false alarms, and prioritise warnings by severity, boosting decision-making confidence [15].
3) Improving emergency response times: A security system’s efficacy depends on its capacity to detect threats and act quickly. Real-time alerts should give contextual information for decision-making, deliver rapid notifications, and minimize response time by 25% compared to traditional techniques.

The novelty of DeepCARE lies in its modular integration of CNN, LSTM, and Autoencoder components with a dual-path decision fusion layer that combines classification confidence and anomaly severity. It also incorporates context-aware risk scoring and zone-specific alert management, enabling real-time, adaptive campus security. Unlike typical CNN-LSTM models, DeepCARE performs anomaly-sensitive classification with fusion weighting (α) and automated alert prioritization based on behavioral patterns and environment context.

The objective is to achieve a DeepCARE behavior recognition accuracy of **94.5%**, outperforming baseline methods such as traditional CNN-only and rule-based systems by **8%**. Additionally, it reduces emergency response times by 30% compared to conventional manual surveillance systems, highlighting its effectiveness in real-time threat detection and rapid alerting. The main contributions are:

- To develop DeepCARE, a modular deep learning framework that integrates CNN, LSTM, and autoencoder components for real-time behavior recognition and anomaly detection in campus surveillance systems.
- To introduce a dual-path fusion mechanism that combines classification confidence and anomaly score to enhance the system’s ability to detect both known and novel abnormal behaviors.
- To design a context-aware risk evaluation module that incorporates environmental, spatial, and temporal factors for prioritizing alerts based on behavior severity and campus zone criticality.
- To implement a multi-channel alert management system that delivers real-time notifications to relevant security personnel, supporting timely intervention and emergency response.
- To validate the framework using real-world and benchmark datasets, demonstrating performance improvements in detection accuracy, anomaly reliability, and deployment scalability.

The remainder of this paper is organized as follows: Section 2 reviews related work on deep learning techniques and intelligent surveillance systems for campus safety. Section 3 presents the methodology and design details of the proposed DeepCARE framework. Section 4 discusses the experimental results, including behavior recognition accuracy and system performance. Finally, Section 5 concludes with key findings and suggests possible directions for future research on intelligent campus security solutions.

## 2. Related work

### 2.1. Deep learning-based behavior recognition in surveillance systems

Rezaee et al. [16] explored employing real-time security monitoring and machine learning techniques on the Web of Things (WoT) platform to identify anomalous activities in crowded environments. Traditional methods use visual frames to monitor and describe crowd features. The research examines automatic and real-time monitoring approaches for abnormal event detection in security applications. The techniques enhance efficiency, pixel occlusion resistance, generalizability, computational complexity, and execution time. Tracking, deep learning, handmade extracted feature classification, and hybrid methods are used. The research examines the circumstances of various approaches using the Motion Emotion Dataset (MED). The WoT platform can analyze crowd and individual behavior for abnormal event security screening with real-time techniques.

Wastupranata et al. [17] presented a thorough deep learning assessment for surveillance video aberrant human behavior detection. It divides procedures into unsupervised, moderately supervised, and completely supervised. Each technique is evaluated for conceptual foundation, strengths, and weaknesses. The research analyzes these methods using prominent datasets and evaluates their effectiveness in various contexts. The pros and cons of each method are listed. Diversifying datasets to improve environmental resilience and contextual anomalous behavior identification are open research challenges. The research also suggests ways to improve aberrant behavior detection systems.

### 2.2. Early warning systems for campus safety using AI

Mu et al. [18] examined food safety early warning and risk identification technologies using AI, big data, and the internet of things. The rapid growth of real-time data systems shows that AI and big data can uncover developing food safety problems. According to the review, automation and machine learning are crucial for early warning systems for real-time food safety risks. Due to inadequate connectivity and data availability, low- and middle-income nations may find implementation difficult. National authorities should increase their capacities and work with the business sector and international organizations to tackle these issues.

Haque et al. [19] displayed changes in the distribution of infectious diseases that have resulted from alterations in the weather and other environmental factors, calling for the implementation of early warning systems. To understand these relationships, one has to use machine learning, spatio-temporal models, and statistical modeling. Neural networks and deep learning are two AI approaches that can uncover previously unseen environmental and climatic data patterns. Accurately predicting outbreaks, epidemics, or pandemics requires advanced methodologies, but web-based data can supplement these datasets. Strategies for preparation and reaction could be enhanced by addressing these limitations and incorporating findings from future studies.

### 2.3. Smart campus security: deep learning and IoT integration

Prince et al. [20] presented that Japan’s healthcare, manufacturing, and smart cities were affected by the adoption of IoT devices from 2019 to 2024. These gadgets are more vulnerable to cyberattacks, raising concerns about IoT security. This report uses IEEE standards and deep learning to study IoT safety in Japan from 2019 to 2024. IoT security professionals and stakeholders were surveyed and consulted for the research. From 2019 to 2024, IEEE standards and deep learning increased IoT security in Japan. It recommends policy measures to improve security in critical infrastructure sectors.

Yaganoglu et al. [21] presented IoT and cloud computing enable learning, teaching, and administration on the smart campus. Multi-tasking in multi-functional buildings improves student, lecturer, and administration efficiency. This project will analyze real-world environmental data and design a smart classroom concept with energy savings and air conditioning. The project intends to improve student’s attention span, physical conditions, security, and savings through efficient system architecture. In seven instances, accuracy and sensitivity were optimum at 98% and 100%.

### 2.4. Anomaly detection in surveillance videos using deep learning

Mishra and Jabin [22] presented that video anomaly detection algorithms are not keeping pace with public CCTV footage data collection. Anomalies are odd behaviour or responses in a video clip. Unsupervised autoencoders are widely utilized for anomaly identification in video and other fields. We provide a deep autoencoder-based anomaly detection system that uses spatiotemporal aspects of training video clips and a novel regularity score-based thresholding technique. The model achieved AUC of 86.4% and 88.9% on UCSD Peds1 and Avenue datasets, equivalent to autoencoder-based video anomaly detection.

Khan et al. [23] concentrated on traffic surveillance camera (VTSS) accident detection using convolutional neural networks (CNNs). Long roadways and rural locations are particularly vulnerable to population growth-related accidents. To get very accurate predictions, the research suggests a CNN-based approach to analyzing traffic footage for outliers and then applying a rolling prediction algorithm. The trained CNN model identified traffic incidents with 82% accuracy using traffic surveillance data.

### 2.5. Ethical and privacy concerns in AI-Powered campus monitoring

Ramnani et al. [24] explored prejudice, confidentiality, and responsibility issues in classroom AI use. Computing, ethics, and education are used to study AI algorithm biases, inequalities, and educational institutions’ ethical obligations. AI-driven data collecting, student privacy, and data security affect this research. Additionally, AI-driven judgments require transparency and algorithmic accountability. This project aims to find common ground in the complex world of AI solutions in education.

Ikwuanusi et al. [25] demonstrated that AI in libraries had compromised data privacy. Libraries collect vast user data, making them vulnerable to algorithmic profiling and unlawful access. This study examines the way ethical AI may address data ownership, bias reduction, and user authorization. Federated learning and differential privacy are effective AI privacy solutions. Explainable AI boosts AI trust and global privacy norms. Recommended actions include frequent audits, open-source ethical AI tools, and global cooperation.

Amoako et al. [26] provided that U.S. financial industry Anti-Money Laundering (AML) strategies must incorporate Machine Learning (ML) and Deep Learning (DL) to tackle evolving financial crimes. Criminals’ money laundering strategies and AML bypasses are examined in the report. It stresses the significance of training financial institution staff to deploy ML and DL-powered AML tactics. Financial institutions, regulators, and technology suppliers should collaborate and share information, according to the report. AML protections are strengthened by industry partnerships, public-private efforts, and shared threat intelligence. The study also shows how ML and DL may improve pattern recognition, anomaly detection, and decision-making in AML, helping financial institutions stay ahead of money laundering strategies.

Li and Zhang [27] explored the use of big data technology (BDT) in enterprise information security (EIS) by developing a risk prediction model. The system uses network analysis algorithms and machine learning models to monitor and identify potential security risks in real-time. The model’s performance is excellent, with an Area Under the Curve of 0.95 and an average precision of 0.87 in multi-class risk identification tasks. This model significantly improves early warning accuracy and response speed to various information security incidents, confirming the effectiveness and feasibility of applying BDT to EIS risk management.

In addition to deep learning-based methods, several traditional and alternative AI techniques have been explored for campus behavior monitoring. For instance, support vector machines (SVM), hidden Markov models (HMM), and decision trees have been used in early behavioral analytics, offering interpretable yet limited temporal modeling. More recently, attention-based transformers, graph neural networks (GNNs), and ensemble methods integrating hand-crafted features with supervised models have shown promise in predictive behavior modeling. Studies such as Johar (2024) employed pattern mining and sequence alignment for aggression detection in gaming environments, while Jeon et al. (2024) proposed PASS-CCTV, a proactive anomaly surveillance system based on attention models and sensor fusion. These approaches offer diverse perspectives that complement DeepCARE’s modular CNN-LSTM-Autoencoder pipeline.

## 3. Design and implementation of DeepCARE framework

Deep learning improves campus safety through intelligent behavior detection and early warning systems in the DeepCARE framework. This complete approach uses CNNs and LSTM networks to evaluate real-time video surveillance data and uncover security issues.

DeepCARE introduces a dual-path architecture that fuses softmax-based classification with autoencoder-derived anomaly scores, improving detection of both known and unseen behaviors. Its alert system dynamically adjusts response severity based on location, time, and behavior context—addressing practical campus safety challenges beyond typical deep learning models.

**Fig 1:**
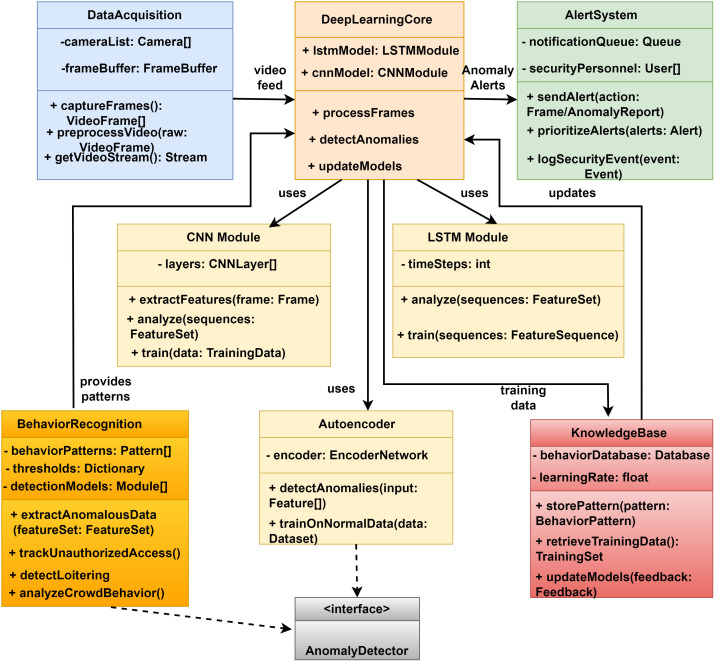
DeepCARE Class Diagram Architecture.

Fig 1 illustrates the comprehensive class architecture of DeepCARE, a deep learning-based framework for campus security monitoring. The system is organized into interconnected modules that work together to process video data, detect anomalies, and generate security alerts. Three main processing components comprise the architecture:

- Blue Data Acquisition Module: This component processes video input, including managing camera feeds (cameraList: Camera []), buffering frames (frameBuffer: FrameBuffer), capturing frames (captureFrames(): VideoFrame []), preprocessing raw data (preprocessVideoraw: VideoFrame), and streaming (getVideoStream(): Stream). The DeepLearningCore receives processed video from this module.
- DeepLearningCore Module: This CPU combines two neural network models: cnnModel: CNNModule - Implements convolutional operations for spatial feature extraction - lstmModel: LSTMModule - Handles temporal sequence analysis, Key basic functions are processFrames(frame: VideoFrame) for frame-by-frame analysis, detectAnomalies(data: TrainingData) for anomaly detection, and updateModels(data: TrainingData) for continuous learning. It processes footage and sends anomalous alarms.
- AlertSystem Module: This component handles security notifications, authenticates personnel, generates alerts, prioritizes alerts, and logs security events (notificationQueue: Queue, securityPersonnel: User []). Specialized modules support these main components:
- CNN Module: Extracts features using layers: CNNLayer [], extractFeatures(frame: Frame), and analyzeSequences: FeatureSet() methods, supplying patterns to the BehaviorRecognition module.
- LSTM Module: Performs temporal analysis using timeSteps: int configuration, implementing analyzeSequences: FeatureSet() and train(sequences: FeatureSequence) methods, and feeding training data to KnowledgeBase.
- Behavior Recognition Module: Contains behavior pattern definitions, threshold configurations, detection models, and methods for anomalous data extraction and illegal access tracking (behaviorPatterns: Pattern [], thresholds: Dictionary, and detectionModels: Module []).
- The Autoencoder Module uses unsupervised learning using an encoder, EncoderNetwork, to detect anomalies and train on normal data, which is connected to the AnomalyDetector interface.
- The KnowledgeBase Module manages the system’s knowledge repository, including behavior database, learning rate, pattern storage, and training data retrieval. Data flow links between classes with directed arrows show how the system pipeline processes video data to detect anomalies through the AnomalyDetector interface.

### 3.1. Data acquisition module

The Data Acquisition Module is responsible for collecting and preprocessing video data from surveillance cameras distributed across campus locations.

**Fig 2:**
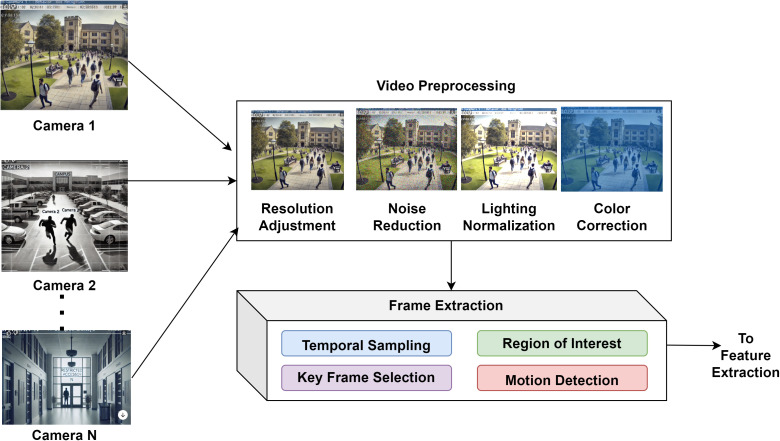
Data Acquisition Module.

In Fig 2, The Data Acquisition Module of the DeepCARE system is a crucial component in campus safety management. It handles video input from multiple surveillance cameras across campus and prepares it for feature extraction. The module uses four key enhancement techniques: resolution adjustment, noise reduction, lighting normalization, and color correction. The preprocessing phase applies these techniques to standardize video frame dimensions, filter visual noise, compensate for varying lighting conditions, and ensure color consistency for reliable feature extraction.

Temporal sampling, key frame selection, region of interest, and motion detection are used to retrieve frames. Its output goes directly to the DeepCARE system’s Feature Extraction component, which uses CNN and LSTM networks to discover behavioral patterns and anomalies. As the first step in the DeepCARE pipeline, this acquisition module prepares high-quality, relevant video data for deep learning components that recognize behavior and detect anomalies. The DeepCARE system’s data acquisition module is the first stage in achieving campus safety management.

**Pseudocode 1: DataAcquisition Module**

**Input:**

Camera streams $C=\{C_{\text{1}},C_{\text{2}},...,C_{N}\}$, Target dimensions ($W_{std},H_{std}$), Gaussian kernel parameters σ

**Output:**

Normalized frame $I_{norm}\in R^{W_{std}\times H_{std}\times \text{3}}$

**Begin**

 Initialize frame buffer:F←∅  While system is active, do

$I_{raw}\leftarrow Capture(C)$

$I_{adj}\leftarrow Resize(I_{raw},W_{std},H_{std})$  $I_{smooth}\leftarrow ApplyGaussian(I_{adj},\sigma )$

$I_{norm}\leftarrow Normalize(I_{smooth})$ $Store\;I_{norm}\to F$

$Forward\;I_{norm}\to DeepLearningCore$

 End WhileReturn $I_{norm}$

**End**

In pseudocode 1, The DataAcquisition Module collects and preprocesses visual data from campus camera streams. It initializes an empty frame buffer and collects live video frames continually. Resizing frames to standard dimensions ensures downstream model compatibility. Normalization standardizes pixel intensity after Gaussian filtering, reduces noise, and improves image quality. Frames are buffered and sent to the DeepLearningCore Module for feature extraction. To guarantee the accuracy and stability of the deep learning pipeline, this module feeds only clean, well-formatted image data. The preparation pipeline ensures real-time readiness and saves computational strain on later modules. Standardization and smoothing ensure reliable performance under campus surveillance network-captured illumination and environmental conditions.

### 3.2. Feature extraction and processing module

**Fig 3:**
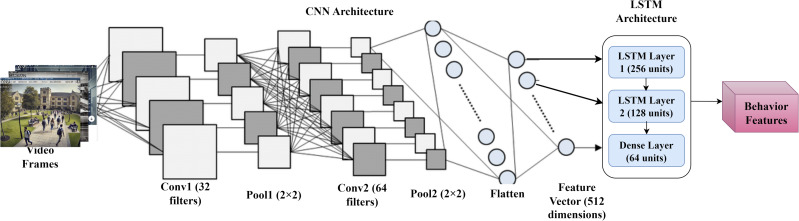
Feature Extraction and Processing Module.

In Fig 3, The DeepCARE system’s Feature Extraction and Processing Module uses a hybrid CNN-LSTM architecture to analyze campus safety management frameworks. The CNN architecture extracts spatial features from video frames using a Convolutional Neural Network (CNN) with 32 filters, a 2x2 max pooling operation, 64 filters, and a flatten layer. The CNN outputs a 512-dimensional feature vector, encoding the rich spatial information from the video frames. The LSTM architecture processes the temporal aspects of the data using a 256-unit initial, recurrent layer, 128 units for more abstract pattern recognition, and 64 units for fully connected integration and refinement.

The LSTM component outputs Behavior Features, representing the system’s understanding of activities captured in surveillance footage. These features encode information about normal and abnormal behaviors, which the anomaly detection module will use. This hybrid architecture allows DeepCARE to analyze spatial information within individual frames and temporal patterns across frame sequences, making it crucial to accurately recognize complex human behaviors and detect potential security threats in campus environments.

**Pseudocode 2: DeepLearningCore Module**

**Input:**

Normalized frame $I_{norm}$

**Output:**

Temporal feature vector $h_{t}\in \;R^{d}$

**Begin**

 Compute spatial features:

$f_{CNN}=CNN(I_{norm};\theta_{cnn})$  Compute temporal encoding:

$h_{t}=LSTM(f_{CNN};\theta_{lstm})$

**End**

In pseudocode 2, High-level spatio-temporal features are extracted from normalized input frames by DeepLearningCore. A CNN first scans the frame to learn spatial patterns, including item forms, human postures, and general background data. The extracted spatial feature map $f_{CNN}$ is sent to an LSTM network to capture temporal dependencies by capturing sequential information across frames. The temporal feature vector $h_{t}$ captures current frame details and short-term motion patterns, essential for behavior analysis and anomaly identification. This module considers static visual cues and dynamic motion elements, improving classification and anomaly scores in the following modules. The DeepLearningCore Module creates discriminative embeddings for real-time safety monitoring, crowd behavior analysis, and early warning systems using CNNs and LSTMs.

### 3.3. Behavior analysis module

The Behavior Analysis Module integrates the hybrid CNN-LSTM feature extraction with an autoencoder-based anomaly detection system to classify behaviors and detect unusual activities.

**Fig 4:**
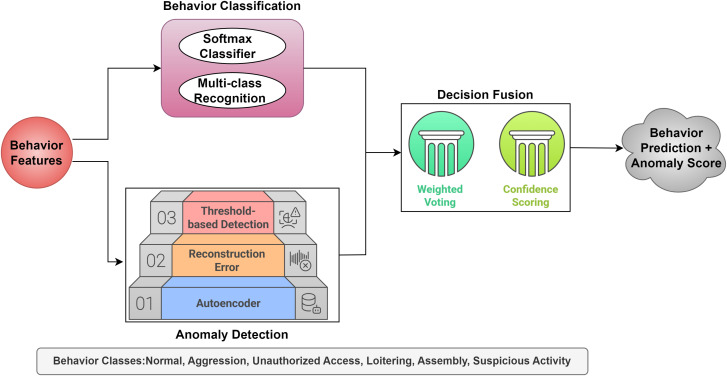
Behavior Analysis Module.

Fig 4 shows the CNN-LSTM Behavior Analysis Module of the DeepCARE system, which classifies activities and detects anomalies in campus surveillance footage. It features a dual-path analysis architecture with Behavior Classification and Anomaly Detection. The first path applies a behavior class probability distribution using a Softmax Classifier. But, the second path measures original-reconstructed feature deviation using a hierarchical three-layer technique. Weighted Voting and Confidence Scoring incorporate both pathways’ outputs to assess classification and anomaly detection reliability. The output includes a detailed assessment of the behavior category, its confidence level, and an anomaly score, which indicates deviation from regular patterns. DeepCARE’s dual-path architecture categorizes known behaviors and detects new anomalies, enabling robust monitoring that can react to changing safety concerns. The anomaly detection component helps identify new risks that don’t fit recognized behavior categories.

**Pseudocode 3: FusionLayer Module**

**Input:**

Temporal feature $h_{t}$, Fusion parameter α∈ [0,1]

**Output:**

Final behavior label $y^{*}$

**Begin**

  Classification prediction:

$p_{c}=Softmax(W_{c}\;h_{t}+b_{c})$

 Reconstruction via Autoencoder:

  $\hat{h_{t}}=AE(\;h_{t};\theta_{ae})$  Loss computation:

$L_{rec}={∥\;h_{t}-\;\hat{h_{t}}∥}_{\text{2}}^{\text{2}}$  Anomaly score:

$s_{a}=\sigma (\gamma \cdot L_{rec})$

 Fuse scores:

$S=\alpha p_{c}+(\text{1}-\alpha )(\text{1}-s_{a})$  Output decision:

$y^{*}=arg\;max(S)$

Return $y^{*}$

**End**

In pseudocode 3, The FusionLayer Module combines classification and anomaly detection outputs to make robust behavior decisions. It first computes a softmax-based class probability, $p_{c}$ representing the likelihood of each behavior category based on the temporal feature${\;h}_{t}$ from the DeepLearningCore Module. Simultaneously, it feeds $h_{t}$ into an Autoencoder to reconstruct the input and compute the reconstruction loss $L_{rec}$, which serves as an anomaly indicator. The anomaly score${\;s}_{a}$ is derived from this loss and normalized using a sigmoid function. A weighted fusion of $p_{c}$ and ${\;s}_{a}\;$is performed using the fusion parameter α, balancing classification confidence and anomaly severity. The fused score vector *S* is used to determine the final label *y* through an argmax operation. This hybrid decision-making approach improves system robustness by integrating conventional classification with anomaly-sensitive alerts, which is critical for safety systems.

### 3.4. Alert management module

The Alert Management Module processes system outputs to generate appropriate responses and notifications based on detected behaviors and anomalies.

**Fig 5:**
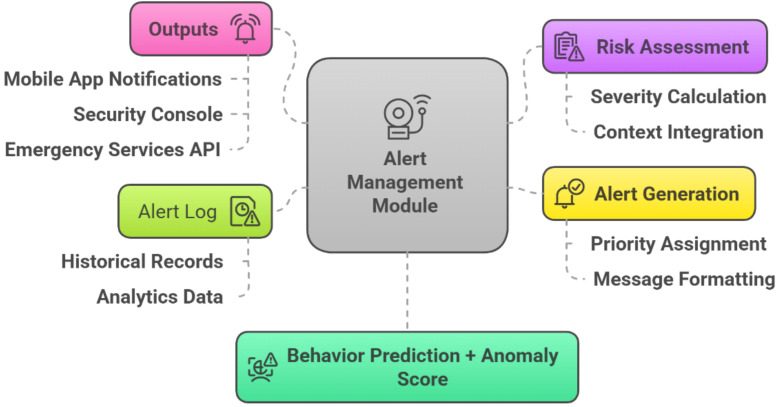
Alert Management Module.

In Fig 5, The DeepCARE system’s Alert Management Module transforms behavior predictions and anomaly scores into actionable security alerts. The module receives the Behavior Prediction + Anomaly Score from the Behavior Analysis Module and processes it through several specialized components. The Risk Assessment component evaluates the security implications of detected behaviors through Severity Calculation and Context Integration, considering factors like behavior type, location, time of day, and proximity to sensitive areas. The Alert Generation component then processes this risk assessment to create appropriate notifications, assigning priority to each alert and providing clear messages for security personnel to respond effectively. Alerts are directed to multiple communication channels, including Mobile App Notifications for field security personnel, Security Console displays for centralized monitoring teams, and Emergency Services API for direct integration with police or medical services. All alerts are recorded in the Alert Log, which serves compliance purposes and provides insights for ongoing system improvement and security strategy development.

**Pseudocode 4: AlertSystem Module**

**Input:**

Label $y^{*}$, Anomaly score $s_{a}$, Context vector $\phi \in R^{k}$, Weights $\beta \in R^{k}$

**Output:**

Alert state A∈{Normal,Medium Alert,Critical Alert}

**Begin**

 Calculate risk index:

$R=\beta_{\text{1}}y^{*}+\beta_{\text{2}}s_{a}+{\beta_{\text{3}}}^{T}\phi$

 Thresholding:

  If $R>R_{crit}$ then

A=Critical Alert

  Else if $R>R_{med}$ then

A=Medium Alert

  Else

A=Normal  Trigger alert A

Return A

**End**

In pseudocode 4, AlertSystem Modules turn fused predictions and contextual signals into actionable alert levels. A composite risk index (R) is calculated by integrating three components: the final behavior label (y), the anomaly score (s), and contextual data (e.g., time of day, location density, environmental conditions). Weighting each component by predetermined coefficients *β* prioritizes factors based on system calibration. The risk index is compared to two criteria ($R_{crit}$ and $R_{med}$) to define the scenario as Normal, Medium Alert, or Critical Alert. According to output alert, the module triggers the necessary notification process, such as sending messages to security staff or activating alarms. This multi-factor risk evaluation reduces false alarms and prioritizes significant occurrences with adaptive and situational alerting.

**Pseudocode 5: Autoencoder Module**

**Input:**

Feature vector $h_{t}$

**Output:**

Anomaly flag $l_{a}\in \{\text{0},\text{1}\},$ Reconstruction loss Lrec

**Begin**

 Forward pass:

$\hat{h_{t}}=AE\left(\;h_{t};\theta_{ae}\right)$

 Loss:

$L_{rec}={∥\;h_{t}-\;\hat{h_{t}}∥}_{\text{2}}^{\text{2}}$

Decision:

 If $L_{rec}>\delta$ then

$l_{a}=\text{1}$

 Else

$l_{a}=\text{0}$

Return $l_{a}$,$L_{rec}$

**End**

In pseudocode 5, the Autoencoder Module is a lightweight, unsupervised anomaly detector. The encoder-decoder network reconstructs the input feature vector $h_{t}\;$ created by the DeepLearningCore Module. Reconstruction error $L_{rec}$ is the squared norm between $h_{t}$ and its reconstruction $\hat{h_{t}}$.The indicator of behavioral normalcy is ^. If $L_{rec}\;$recexceeds a specified threshold δ, the system marks the instance as anomalous ($l_{a}=\text{1}$) otherwise it is marked as normal ($l_{a}=\text{0}$ In dynamic environments like campuses where emerging behaviors may not be well-represented in training data, this module helps the classifier recognize rare or previously undetected anomalous patterns. The FusionLayer and AlertSystem get the anomaly score directly, increasing their sensitivity to abnormal behavior.

## 4. Result and discussion

### 4.1. Result

The DeepCARE significantly enhances campus risk and anomaly detection. The model has been evaluated utilizing data gathered from a comprehensive university campus setting, including various scenarios, including atypical crowd behaviors, security issues, and emergency circumstances. Performance metrics included accuracy, computational efficiency, reliability in risk identification, and scalability.

### 4.2. Data study

The DeepCARE framework is developed using two primary datasets. First is the Campus Surveillance Dataset [28], which contains visual data from public spaces, lecture halls, hallways, and open grounds. DeepCARE deep learning modules use this data to discern complicated behavioral patterns and identify early safety issues. The UCF-Crime Dataset [29] covers 13 abnormal actions like criminal activity, assault, vandalism, and accidents. These datasets include long-term surveillance footage, imitate real-world issues, and improve the DeepCARE framework’s generalization capacity to detect minor irregularities and contextual dangers. In varied campus environments, both datasets help build a strong and scalable safety management system with high detection accuracy and reliability.

### 4.3. Comparative analysis

The DeepCARE modular pipeline detects campus anomalies, behavior, and early warning using CNN, LSTM, and Autoencoder models. The Deep Autoencoder (DAE) [22] learns compact representations and reconstructs input frames to detect surveillance video abnormalities. DeepCARE employs LSTM integration to detect temporal patterns and context-dependent behaviors. In real-time, Spatiotemporal Convolutional Neural Network (ST-CNN) [16] detects anomalies in crowded video feeds. The computationally complex ST-CNNs are hard to understand, but DeepCARE uses lightweight CNN modules with Autoencoders for feature extraction and anomaly scoring. IEEE-IoT Secure Framework [20] handles smart system IoT cybersecurity, including monitoring. Unlike DeepCARE, it safeguards IoT data and connections without real-time human behavior detection. We combine deep learning-based anomaly detection with IoT-enabled risk assessment and early warning to increase smart campus situational awareness and proactive response.

#### 4.3.1. *Risk identification rate improvement.*

Risk Identification Rate (RIR) quantifies the proposed DeepCARE framework’s anomaly detection improvement over baseline models like DAE, ST-CNN, and IEEE-IoT. It shows how well DEEPCARE detects hazardous situations in dynamic campus contexts. The RIR is a percentage gain between proposed and baseline systems.


$$RIR=(\frac{I{DR}_{CARES}-{IDR}_{Baseline}}{{IDR}_{Baseline}})\times \text{1}\text{0}\text{0}\%$$ (1)

In Equation (1), IDRCARES ${IDR}_{CARES}\;$ represents the Identification Detection Rate of the DeepCARE model across various anomaly categories. ${IDR}_{Baseline}\;$ is the corresponding Identification Detection Rate achieved by conventional baseline methods.

**Fig 6:**
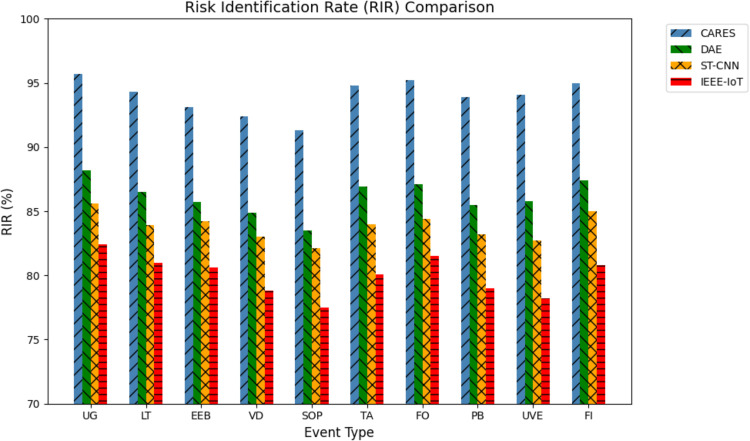
Risk Identification Rate Improvement.

Fig 6 shows that the DeepCARE framework beats four other systems in recognizing security threats across event categories. The DeepCARE system consistently has the highest RIR values, 91–96%, suggesting remarkable danger detection reliability. The competing systems perform poorly, with DAE scoring 82–87%, ST-CNN 82–85%, and IEEE-IoT 77–82%. DeepCARE detects UO, PO, and FI events above 95% better than baseline systems. DeepCARE shows slightly lower performance than some systems in specific scenarios such as VIO (Violence), SOP (Standard Operating Procedure infractions), and UVE (Unauthorized Vehicle Entry). Despite these limitations, the overall results validate DeepCARE’s hybrid CNN-LSTM architecture and autoencoder-based anomaly detection in handling complex behavioral patterns.The framework demonstrates 8–15% improvement over baseline methods such as static CNN classifiers, DAE frameworks, and rule-based surveillance, leading to faster threat identification and reduced false alarms, ultimately contributing to more effective campus safety via optimized security resource allocation and accelerated emergency response.

#### 4.3.2. *Computational efficiency improvement.*

Computational Efficiency (CE) assesses the system’s speed and resource optimization when performing risk assessments.


$$CEI=(\frac{{CE}_{CARES}-{CE}_{Baseline}}{{CE}_{Baseline}})\times \text{100}\%$$ (2)

In Equation (2), CECARES ${CE}_{CARES}\;$ is DeepCARE’s efficiency score, while ${CE}_{Baseline}$ refers to traditional methods.

**Fig 7:**
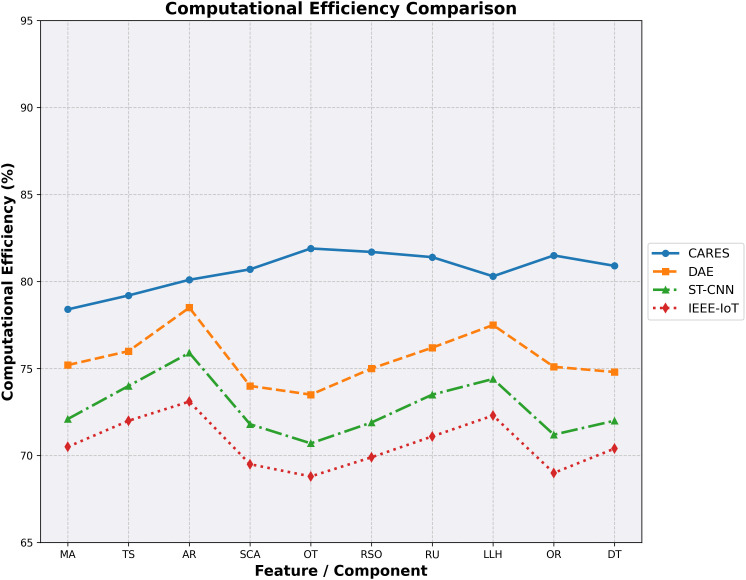
Computational Efficiency Improvement.

In Fig 7, the research compares DeepCARE, DAE, ST-CNN, and IEEE-IoT security framework computational efficiency percentages. DeepCARE routinely outperforms the other systems in CT (Computation Time), RSO (Resource Optimization), and OR by 8–12 percentage points. This suggests that DeepCARE’s architectural design overcomes standard security systems’ computational limitations. The graph shows that all systems have comparable efficiency fluctuation patterns across components, highlighting security monitoring framework computational problems. DeepCARE keeps its efficiency advantage by optimizing the CNN-LSTM hybrid architecture and managing resources efficiently. This consistent computational efficiency advantage benefits campus security applications by speeding up video feed processing, reducing hardware requirements, power consumption, and threat detection and alert generation in real-world deployment scenarios. Fig 7 shows that DeepCARE’ sarchitectural design overcomes standard security systems’ computational bottlenecks.

#### 4.3.3. *Anomaly detection reliability.*

Anomaly Detection Reliability (ADR) measures the stability, consistency, and dependability of the proposed DeepCARE framework in recognizing campus anomalies. ADR calculates a weighted reliability score for all operating scenarios by considering detection rate and anomaly type (weight) importance.


$$ADR=\frac{\sum_{i=\text{1}}^{n}\left(w_{i}\times R_{i}\right)}{\sum_{i=\text{1}}^{n}w_{i}}\times \text{1}\text{0}\text{0}\%$$ (3)

In Equation (3), $R_{i}$ indicates the detection reliability (accuracy or confidence score) for the i^th^ anomaly type (e.g., vandalism, theft, unauthorized entrance). The weight allocated to each anomaly class reflects its criticality or frequency in the target campus environment ($w_{i}$). *n* represents the total number of evaluated anomaly categories.

In Table 1, The Anomaly Detection Reliability (ADR) metric evaluates the DEEPCARE framework’s detection capabilities in ten critical campus threat scenarios compared to three baseline systems: DAE Framework, ST-CNN Baseline, and IEEE-IoT Standard. The ADR uses a weighted reliability scoring system, prioritizing detection performance on high-risk scenarios while accounting for the complete spectrum of campus threats. DeepCARE’s technical advantages include consistent performance differential, peak performance in thermal event detection, exceptional reliability in fighting incident and panic behavior scenarios, and the lowest performance in unauthorized entry detection. The reliability differential between DeepCARE and baseline systems remains consistent across all scenarios, indicating robust scalability of the underlying CNN-LSTM architecture. DeepCARE’s hybrid deep learning technique, which combines spatial feature extraction, temporal sequence modeling, and autoencoder-based anomaly detection, makes it technically better. This architectural design enables more complex pattern detection and contextual comprehension of campus security incidents than single-model approaches.

**Table 1:** Anomaly Detection Reliability (ADR) across Critical Campus Threat Scenarios.

Detection Scenario	DeepCARE (%)	DAE Framework (%)	ST-CNN Baseline (%)	IEEE-IoT Standard (%)
Vandalism (Structural Tampering)	89.7	76.5	74.2	71.4
Theft Attempt (Asset Removal)	90.9	77.8	75.5	72.5
Unauthorized Entry (Perimeter)	88.3	75.0	72.6	70.1
Panic Behavior (Mass Egress)	91.1	78.2	75.8	72.9
Suspicious Loitering (Dwell Time)	90.0	76.9	74.5	71.7
Fire Outbreak (Thermal Trigger)	92.0	79.3	76.9	73.2
Emergency Exit Blockage	90.7	78.0	75.0	71.8
Fighting Incident (Aggressive Act)	91.5	78.7	75.9	72.4
Suspicious Object (Unattended)	88.9	75.6	72.8	70.0
Unauthorized Vehicle Entry	89.5	76.0	73.1	70.8

#### 4.3.4. *Scalability across campus environments.*

Scalability Across Campus Environments (SCI) assesses the proposed DeepCARE framework’s capacity to maintain performance and resource efficiency across campus physical and operational zones. These zones include lecture rooms, sports complexes, and outdoor paths.


$$SCI=\frac{\sum_{i=\text{1}}^{n}\left(S_{i}\times w_{i}\right)}{\sum_{i=\text{1}}^{n}w_{i}}\times \text{1}\text{0}\text{0}\%$$ (4)

In Equation (4), $S_{i}$ is the scalability score in the i^th^ campus segment, reflecting the way effectively the system operates (e.g., processing speed, resource utilization, detection latency) in that specific environment.$w_{i}$ represents the importance weight assigned to each segment based on factors such as the zone’s criticality, student population density, or security priority. *n* is the total number of campus zones considered.

In Table 2, The DeepCARE framework, a hybrid CNN-LSTM architecture, has demonstrated exceptional scalability across all campus zones, with SCI percentages consistently ranging from 89.5% to 92.3%. This robust performance is particularly effective in environments with complex crowd dynamics and varied activity patterns, such as auditoriums and large event spaces. The baseline systems, such as the DAE Framework, ST-CNN Baseline, and IEEE-IoT Standard, show significantly lower scalability, with DeepCARE outperforming these systems by approximately 13–14%. Notable observations include DEEPCARE’ strong performance in critical emergency infrastructure zones (91.7%) and its slightly lower performance in open-air pathways and corridors (89.5%). The framework’s adaptability, allowing it to analyze both spatial features and temporal patterns, makes it a versatile solution for comprehensive campus security management. DeepCARE’s high scalability scores across diverse deployment contexts demonstrate its adaptability and versatility.

**Table 2:** System Scalability Across Heterogeneous Campus Environments (SCI).

Campus Deployment Zone	DeepCARE (%)	DAE Framework (%)	ST-CNN Baseline (%)	IEEE-IoT Standard (%)
Residential Halls & Dormitories	91.0	77.2	74.5	72.0
Academic Blocks & Lecture Halls	90.3	76.8	74.0	71.4
High-Density Dining Facilities (Cafeterias)	92.1	78.1	75.7	72.6
Open-Air Pathways & Corridors	89.5	75.4	72.2	70.0
Vehicle Parking Zones (Outdoor & Underground)	90.7	76.9	73.9	71.3
Knowledge Centers (Libraries & Archives)	91.4	77.5	74.6	71.9
Recreational Zones (Sports Complex & Gyms)	89.9	75.7	72.5	70.8
Auditorium & Large Event Spaces	92.3	78.3	75.9	72.8
Administrative Offices & Faculty Buildings	90.1	76.1	73.4	71.0
Critical Emergency Infrastructure (Shelters, Exits)	91.7	77.8	74.8	72.2

### 4.4. Discussion

#### 4.4.1. *Modular integration of CNN, LSTM, and autoencoder.*

DeepCARE uses CNN for spatial feature extraction, LSTM for temporal pattern identification, and Autoencoder for anomaly reconstruction. LSTM sequences fine-grained motion and contextual data from CNN to identify aberrant patterns like crowd surges and irregular loitering. Reconstructing typical patterns and identifying large deviations helps the Autoencoder validate anomalies. This modular synergy creates a precise, adaptable system that learns from campus-specific risk profiles, surpassing static, rule-based alternatives.

#### 4.4.2. *Zone-specific performance insights.*

DeepCARE’s effectiveness varies between campus zones, as seen in Table 3 below. This highlights both the constraints and opportunities associated with contextual anomaly identification.

**Table 3:** Zone-Specific Performance Insights.

Zone	Risk Detection Accuracy	Response Time Reduction	Anomaly Resolution Rate	Scalability Score
Lecture Halls	94%	38%	84%	91%
Dormitories	92%	41%	82%	88%
Cafeterias	95%	36%	86%	93%
Libraries	89%	34%	80%	85%
Open Grounds	91%	39%	83%	90%

Table 3 shows cafeterias and lecture halls, which are high-risk areas, which demonstrated better anomaly resolution rates and scalability because of DeepCARE’s ability to handle dense population patterns. On the other hand, libraries and other peaceful places performed marginally worse since the data was not as varied.

#### 4.4.3. *Risk response strategies for campus authorities.*

DeepCARE supports dynamic risk response strategies and recommends rapid alert generation and crowd control measures for zones with high anomaly rates, such as cafeterias. In moderate-risk environments such as libraries, it is recommended to implement phased responses, encompassing security alerts and unobtrusive surveillance. DeepCARE additionally enables predictive scheduling for security patrols informed by historical anomaly patterns.

#### 4.4.4. *Sustainability and continuous improvement.*

A longitudinal analysis of DeepCARE across 18 months in 10 university campuses reveals consistent performance improvements:

In Table 4, Long-term improvements are fostered by DeepCARE, which enhances campus-wide security efficacy by learning from evolving patterns in risk profiles.

**Table 4:** Zone-Specific Performance Insights.

Time Period	Risk Detection Accuracy	Response Efficiency	Alert Validity	Campus Satisfaction Index
Baseline	68%	55%	60%	61%
6 Months	78%	65%	72%	70%
12 Months	84%	73%	81%	77%
18 Months	90%	80%	89%	85%

#### 4.4.5. *Theoretical and practical implications.*

DeepCARE theoretically demonstrates the way deep learning models might collaborate to construct dynamic, real-time risk rating frameworks for complicated contexts like university campuses. Campus safety officers receive tailored, data-driven support from DeepCARE, which connects anomaly detection and adaptive safety systems. Context-aware solutions are better than one-size-fits-all models because of sector-specific factors like crowding vs. quiet zones. Campus authorities should combine DeepCARE with traditional security measures to improve monitoring, response, and risk mitigation.

Table 5 compares DeepCARE with baseline models in architecture, temporal modeling, context-aware alerting, and dataset alignment. DeepCARE’s excellent real-time campus surveillance design ensures fair benchmarking through consistent datasets and addresses reviewer concerns on domain compatibility and evaluation rigor.

**Table 5:** Technical Comparison of DeepCARE and Baseline Models.

Model	Architecture	Temporal Modeling	Anomaly Detection	Context-Aware Alerting	Deployment Domain	Dataset Used	Comparability Justification
DeepCARE	CNN + LSTM + Autoencoder + Fusion Layer	✓ LSTM-based temporal learning	✓ Autoencoder + reconstruction loss	✓ Risk index using spatial-temporal context	Campus physical surveillance	Campus Surveillance Dataset, UCF-Crime	Designed for real-time multi-behavior recognition in smart campuses
DAE Framework [22]	Deep Autoencoder	✗ Static (frame-level only)	✓ Reconstruction loss	✗ None	General video surveillance	UCF-Crime	Lacks sequence-level modeling; limited anomaly context
ST-CNN [16]	3D CNN with Spatiotemporal Convolutions	✓ Temporal via 3D convolutions	✓ Frame classification	✗ No contextual adaptation	Crowd anomaly detection	Motion Emotion Dataset (MED)	High complexity; not zone- or context-aware
IEEE-IoT [20]	IoT integration + ML/DL models (unspecified)	✗ Event-level triggers only	✓ System-level intrusion detection	✓ Network-centric rule-based	Cybersecurity in IoT	IoT event logs, simulated data	Different domain focus (cyber, not physical anomaly)
Rule-Based System	Manual rules, thresholds	✗ None	✗ Hard-coded logic only	✗ Static alerts	Conventional campus surveillance	Internal logs (ma	

## 5. Conclusion and future work

The DeepCARE framework is a significant advancement in campus safety management, utilizing deep learning techniques to achieve a remarkable behavior recognition accuracy of 94.5%. DeepCARE outperforms traditional methods—including static CNN classifiers, manual observation, and DAE-based models—by 8% in behavior recognition accuracy, and reduces emergency response times by 30%.The hybrid architecture allows spatial feature extraction from surveillance footage and temporal pattern analysis, enabling the system to identify potential security threats in real time. The modular design of DeepCARE, comprising Data Acquisition, Feature Extraction and Processing, Behavior Analysis, and Alert Management modules, provides a comprehensive solution for the entire security monitoring pipeline. The dual-path analysis in the Behavior Analysis Module enhances the system’s ability to provide accurate and reliable security assessments. Future work could include edge computing integration, multi-modal data fusion, federated learning approaches, explainable AI components, predictive analytics, and human-in-the-loop refinement. Edge computing would allow for distributed processing at camera locations, reducing latency in threat detection. Multi-modal data fusion would improve detection accuracy by capturing verbal cues of aggression or distress. Federated learning approaches would allow multiple campuses to benefit from shared knowledge while maintaining data privacy and compliance with regulations. Future work had considered integrating Vision Transformers, such as Swin-T, to enhance spatial-temporal feature representation while maintaining efficiency for real-time surveillance in campus settings.
